# Efficacy and safety of sublingual immunotherapy using a combination of *Dermatophagoides pteronyssinus* and *Blomia tropicalis* extracts in patients with allergic rhinitis: A randomized, double-blind, placebo-controlled trial^☆^

**DOI:** 10.1016/j.waojou.2024.101020

**Published:** 2025-01-28

**Authors:** Priscilla Rios Cordeiro Macedo, Priscila Moraes, Luísa Karla Arruda, Fábio Fernandes Morato Castro, Jorge Kalil, Clóvis Eduardo Santos Galvão

**Affiliations:** a Clinical Immunology and Allergy Unit, Hospital das Clínicas, University of São Paulo Medical School, São Paulo, SP, Brazil; b Departments of Medicine, Ribeirão Preto Medical School, University of São Paulo, Ribeirão Preto, SP, Brazil

**Keywords:** Allergen immunotherapy, Allergic rhinitis, Blomia tropicalis, Dermatophagoides pteronyssinus, Sublingual immunotherapy, Drops

## Abstract

**Background:**

Allergen immunotherapy is the only treatment that may modify the natural course of allergic diseases. Sublingual immunotherapy (SLIT) is a promising treatment, especially for children. Few studies currently exist related to optimal dosing for *Blomia tropicalis*.

**Methods:**

This was a double-blind, randomized, placebo-controlled trial of SLIT to treat house dust mite-induced Allergic Rhinitis (AR). A total of 65 patients, ages 12–16 years, were treated for 12 months and randomized into SLIT versus placebo. The SLIT group received a combination of *Dermatophagoides pteronyssinus* and *Blomia tropicalis* allergens. Sensitization was confirmed by skin prick test or serum specific IgE. Total Nasal Symptom Score (TNSS), Rhinoconjunctivitis Quality of Life Questionnaire (RQLQ), current treatment, and need for medication to control symptoms were ascertained during the study. Total serum IgE, serum specific IgE, and IgG4 levels for *Der p* 1 and *Blo t* were assessed at baseline and, 6 and 12 months after treatment.

**Results:**

There was no significant difference in the number of adverse events between groups. The SLIT group showed a significant reduction in antihistamine use to control symptoms (p < 0.0001) compared to placebo. There was no significant change in serum total IgE, serum specific IgE, and IgG4 for either allergen when comparing the SLIT and placebo groups.

**Conclusion:**

After 1 year, SLIT using a dose of 1 mcg of Der p 1/day and 753 UBE of Blo t/day lowered the need for medications for break-through symptoms, with a good safety profile."

## Introduction

House dust mite (HDM) is a major indoor allergen worldwide, frequently triggering nasal symptoms in patients with perennial allergic rhinitis (AR) — a chronic inflammatory disease of the airways.^1^

The treatment of allergic rhinitis involves 3 main strategies: avoiding allergen exposure, using medications to control symptoms, and implementing allergen-specific immunotherapy (AIT).^2^ While avoiding allergens is not always feasible, pharmacotherapy options such as nasal steroids and antihistamines can effectively alleviate symptoms. However, AIT is currently the only causal treatment for allergic rhinitis and serves as a preventive measure for asthma. It represents a promising strategy for early intervention to manage allergic rhinitis. Although the precise action mechanisms are not fully understood, immunotherapy has been widely used to treat allergic diseases, and its effectiveness is well recognized.^3^, ^4^, ^5^, ^6^, ^7^, ^8^

In the field of allergy treatment, there are 2 primary administration routes: subcutaneous immunotherapy (SCIT) and sublingual immunotherapy (SLIT), the last one is known for its safety.^9^ Due to its safety profile, SLIT can be administered at home, except for the first dose, which should be given under clinical supervision.^10^^,^^11^ SLIT is particularly appealing for children because it is painless and can stimulate the regulatory immune response, potentially preventing the development of severe rhinitis and asthma in later life.^12^^,^^13^

There are 2 formulations for SLIT: tablets (either compressed or freeze-dried) and liquid drops. Only SLIT tablet products are approved by the Food and Drug Administration (FDA) in the United States and the European Medicines Agency (EMA), while SLIT drops are used off-label from SCIT extracts.^14^ Unlike SLIT tablets, SLIT drops allow for the treatment of multiple allergen sensitivities simultaneously, similar to SCIT. However, in some regions, such as Brazil, only SLIT drops are available.

Currently, information on dose, regimen, and duration of SLIT is well established for SLIT tablets, mainly for pollens and house dust mites (HDM), more specifically *Dermatophagoides pteronyssinus* (*D. pteronyssinus*) and *Dermatophagoides farinae* (*D. farinae*).^11^^,^^14^^,^^15^ In tropical countries characterized by hot and humid weather, the storage mite *Blomia tropicalis* (*B. tropicalis*) also has clinical relevance, in addition to *D. pteronyssinus* and *D. farinae* species.^16^, ^17^, ^18^ Since most studies with HDM SLIT have been conducted in European and North American countries, the optimal maintenance dose for *B. tropicalis* remains undetermined and varies from trial to trial.^19^, ^20^, ^21^, ^22^, ^23^

In Brazil, the prevalence of sensitization of patients with allergic rhinitis to *Dermatophagoides pteronyssinus* and *Blomia tropicalis* is 83% and 70.3%, respectively, and most of Brazilian patients are sensitized to both allergens simultaneously.^17^ Despite the high prevalence of sensitization to *B. tropicalis* in tropical countries, there are few studies involving the use of extracts for this allergen, either alone or in combination with *Dermatophagoides* sp*.*^19^, ^20^, ^21^, ^22^, ^23^

Given the increasing use of sublingual immunotherapy (SLIT) for treating allergic diseases, particularly allergic rhinitis (AR), and the unavailability of SLIT tablets in our country, as well as the significance of sensitization to *B. tropicalis*, the primary aim of this clinical trial was to evaluate the safety and efficacy of a liquid drop formulation combining extracts of *D. pteronyssinus* and *B. tropicalis*. This study focused on adolescents and adults with house dust mite-induced AR in a Brazilian population, comparing the treatment against placebo over a 12-month period.

## Methods/design

### Study design

**Fig. 1:**
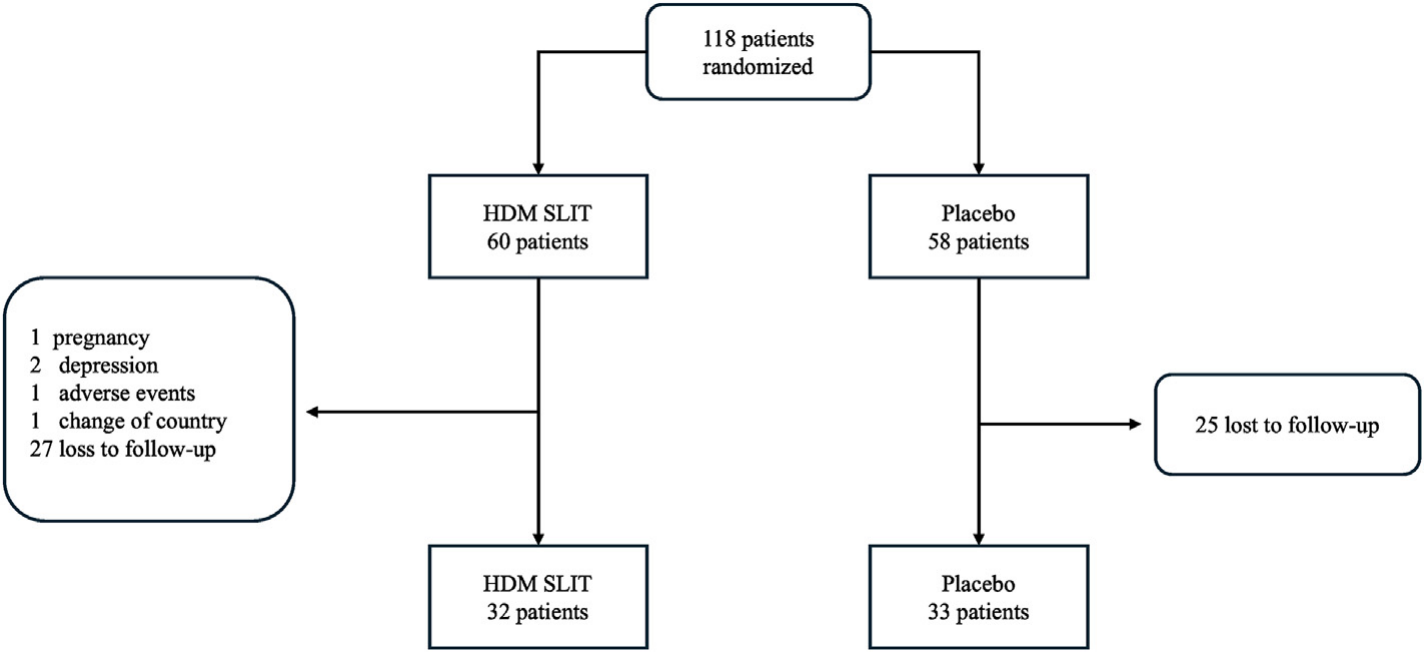
Patient flowchart over 12 months. A total of 118 patients were selected and randomized into 2 groups: treatment group with house dust mite sublingual immunotherapy (HDM SLIT) with *D. pteronyssinus* and *B. tropicalis* extract and placebo group. A total of 65 patients completed the study. Follow-up analysis were conducted in a double-blind fashion and groups were only revealed after completion of all clinical and immunological analyses

This randomaized, double-blind, placebo-controlled trial (RDBPCT) involving patients diagnosed with moderate/severe persistent allergic rhinitis, sensitized to *D. pteronyssinus* and *B. tropicalis*, at a single center, the Hospital of the University of Sao Paulo Medical School. Participants were recruited between July 2017 and January 2019. The trial design is shown in Fig. 1.

A total of 118 subjects received treatment with the combined extract of house dust mite sublingual immunotherapy (HDM SLIT) or with a placebo for 12 months as adjuvant treatment for allergic rhinitis. During the trial, intranasal corticosteroid (budesonide), and antihistamine eye drops (olopatadine) (for those with associated conjunctivitis) were maintained. Oral antihistamines (loratadine) were used as an emergency medication. Data on rhinitis symptoms and medication use were collected during the baseline, 6, and 12 months of treatment. Patients with recurrent rhinosinusitis were screened for an inborn immunity error and anatomical causes (computed tomography of the paranasal sinuses).

### Patients

The participants were selected from those who attended the Allergy, Immunology and Otolaryngology Outpatient Clinics of Hospital das Clinicas of the University of São Paulo Medical School. To be eligible for inclusion in the present study, patients needed to be 12–60 years of age, with moderate/severe persistent allergic rhinitis,^24^ rhinitis symptoms for 2 years or more, and sensitized to *D. pteronyssinus* and *B. tropicalis* defined by positive skin prick tests (≥3 mm) and/or presence of serum immunoglobulin E (IgE) specific to *D. pteronyssinus* and *B. tropicalis* measured by ImmunoCAP® (>0.35 kU/L). Exclusion criteria were pregnancy, persistent asthma,^25^ forced expiratory volume in the first second (FEV1) less than 70%, asthma attack in the past 12 months or need for inhaled corticosteroids, use of immunosuppressors to treat inflammatory diseases, heart, and psychiatric diseases or primary or secondary immunodeficiencies, previous treatment with specific allergen immunotherapy, and sensitization to allergens other than *D. pteronyssinus*, *D. farinae*, and *B. tropicalis* — HDM with clinical relevance.

### Randomization and blinding

Patients were randomly allocated to a treatment with HDM SLIT or placebo and identified with a number. The company's senior manager, who provided the extract for HDM SLIT or placebo, held the secret code for each group until the end of the trial.

### Treatment with SLIT and placebo

The treatment regimen comprised a daily administration of SLIT with combined *D. pteronyssinus* and *B. tropicalis* extracts or placebo delivered by sublingual route, comprising a 1-min holding period and subsequent swallowing.^26^ The HDM SLIT extracts used in the present trial were provided by the International Pharmaceutical Immunology Brazil Inc Inc (IPI-ASAC Brazil) laboratory, licensed in Brazil to prepare, and commercialize the SLIT material produced by ASAC Pharma Inc from lyophilized extracts in Spain.

The vials with the combination of extracts and placebo were labeled only with the bottle number and technical information, including the expiration date, concentration, and batch. Only the laboratory team had access to patient group information during the study period and up until the completion of the results analysis.

There was only 1 extract dilution during all studies (50%:50%). Each vial in the treatment group contained 5 mcg/mL of *Dermatophagoides pteronyssinus* allergen 1 (Der p 1) and 3.765 UBE/mL of *Blomia tropicalis* allergens (Blo t). Dilutions were prepared with double-distilled water containing 50% glycerol. Each drop contained approximately 0.25 mcg of Der p 1 and 188.25 UBE of *B. tropicalis*. The placebo solution was identical to the diluent of the extract, composed of double-distilled water and 50% glycerol.

The first dose was administered under medical supervision with a 30-min observation period following intake. The build-up phase involved administering increasingly larger doses, adding 1 drop per day until reaching 8 drops/day, which contained 2 mcg/day of Der p 1 and 1.506 UBE/day of *B. tropicalis*. Nonetheless, in the first month of the study, 9/20 patients presented dyspnea, wheezing, and cough; from these 9 patients, 7 had intermittent asthma with no need control medication. We chose to maintain the blinding of the study, and for safety reasons, the maintenance dose was reduced to 1 mcg/day of Der p 1 and 753 UBE/day of *B. tropicalis* (4 drops/day) for all patients. All patients received an action plan to treat adverse reactions.

### Clinical and laboratory assessment

All patients underwent clinical assessment each 3 months during the first year of treatment, returning empty vials and receiving new vials of the treatment. Clinical parameters were measured including the need for rescue medication (each 3 months), Total Nasal Symptoms Score (TNSS) (each 3 months), and Rhinoconjunctivitis Quality of Life Questionnaire (RQLQ) (each 6 months).

The TNSS assessed symptom severity of 4 rhinitis symptoms: blocked nose, sneezing, runny nose, and itchy nose, and ranging from 0 to 12 points. The higher score indicates greater severity of symptoms.

To measure quality of life impacts, patients completed the Portuguese version of the Rhinoconjunctivitis Quality of Life Questionnaire (RQLQ). This questionnaire was developed to measure the impact of rhinitis, in the lives of those with rhinoconjunctivitis, and overall scores range from 0 to 6 and, the closer to 0, the better the quality of life.^27^ Change in the RQLQ score of 0.5 or more is recognized as clinically significant, indicating meaningful improvement or deterioration in quality of life over time.^28^ Participants filled out both the TNSS and the RQLQ questionnaires at 3 time points: baseline (T0), after 6 months of treatment (T6), and after 12 months of treatment (T12). These evaluations occurred prior to any clinical assessments by the researchers to ensure that the patients’ self-reported outcomes were unaffected by external evaluations.

Patients were also monitored for loratadine use, an antihistamine, as a rescue medication. They were asked about the frequency of loratadine use over the past week at each visit, providing insight into the effectiveness of the treatment regimen and their ongoing symptom management. Two researchers performed the clinical evaluation during the study period. Patients had unrestricted access to researchers in the case of adverse events and any other questions using a messaging application.

Immunological parameters were assessed at baseline (T0), T6 and T12 using ImmunoCAP™ (Thermo Fisher Scientific, São Paulo, Brazil). The measured markers were total IgE levels and specific IgE and IgG4 levels to Der p 1 and Blo t, in kU/L or kUA/L according to the manufacturer's instructions.

### Primary outcomes

The meaningful within-subject response was a significant reduction of TNSS and a reduction greater than or equal to 20% in the need for medication to control symptoms in the HDM SLIT group during the first 12 months of treatment.

### Secondary outcomes

Compare the RQLQ scores in the HMD SLIT group to the placebo group; evaluate *in vitro* specific immunologic response to treatment and adverse events in both groups.

### Sample size calculation

The sample size was calculated considering the prevalence of rhinitis in this type of treatment at 10%, with a delta of 5%, that is, a significance level of 5%; and a power of 90%. We reached a value of 43 individuals in each group, adding 20% due to possible losses, reaching a final calculation of 51 individuals per study group.

### Ethical aspects

The Declaration of Helsinki conducted the trial of the International Conference on Harmonization Good Clinical Practice guidelines. The Ethics Committee of the Clinical Hospital das Clinicas of the University of Sao Paulo Medical School and the Ethics Committee for Analysis of Research Projects of the Hospital das Clinicas of the University of São Paulo Medical School approved the study (protocol number 1.978.680). Patients or, where appropriate, parents or legal guardians, provided written informed consent. After 1 year of the study, the placebo group also received SLIT treatment.

### Statistical analysis

Continuous variables were described with mean, standard deviation, median, median of absolute deviations from the median, and 95% confidence interval. Continuous data were evaluated using the Shapiro-Wilk test to assess the normality of their distribution and, in case of comparison, the Levene test to assess variance homogeneity.

Comparison of variables by experimental category was performed using the Student's t-test (original or with Welch's modification) or the Wilcoxon-Mann-Whitney test, depending on data distribution type. In situations of more than 2 categorical levels, the ANOVA or Kruskal-Wallis tests were used, depending on the presence or absence of normal distribution. The ANOVA method was used in the repeated measures tests, with a previous test of sphericity and occasional Greenhouse-Geisser or Huynd-Feldt corrections according to the effect size. In cases of normality violation, the Friedman test for repeated non-parametric measures was used.

Categorical variables were described by tabulating frequencies and tested against each other using the chi-square test. In cases where repeated nominal evaluations were tested, the McNemar test was used, and in those where more than 1 categorical variable was tested to determine another variable, the standard logistic model was used with a link variable of the "logit" type, and the iterative weighted least squares method. The significance level used in all tests was 5%, and all tests were performed using the R program version 4.0.5 (03/31/2021) - "Shake and Throw," distributed by "The R Foundation for Statistical Computing Platform."

## Results

### Characteristics of the subjects participating in the study

One hundred and eighteen patients were recruited to the study, and 65 (55%) completed the 12-month treatment. Of these, 34 received SLIT with the combination of *D. pteronyssinus* and *B. tropicalis* extracts, and 31 received placebo (Fig. 1). Table 1 presents the demographic characteristics of the 65 patients who completed the study. During the first year of the study, the dropout rate was 44.9% (Fig. 1). Of these, 84.6% occurred within the first 6 months. There was no dropout difference between the HDM SLIT and placebo groups (p: 0.74). No difference in dropout rates was observed for gender or comorbidity between groups (p > 0.05).

**Table 1:** Baseline demographic and clinical characteristics of patients with allergic rhinitis by treatment group.

Variables	HDM SLIT (n = 32)	Placebo (n = 33)	*p*
Age in years, mean (Min - Max) Age distribution	22.7 (12–48)	21.7 (12–47)	NS^a^
12y- 18y	14 (43.8%)	17 (51.5%)	NS^a^
>18y	18 (56.2%)	16 (48.5%)	NS^a^
Sex			
Male n (%)	17 (53.2%)	12 (36.4%)	NS^a^
Female n (%)	15 (46.9%)	21 (63.6%)	NS^a^
Total nasal symptoms score (TNSS), mean ± DP	6.4 ± 3.02	6.09 ± 2.64	NS^a^
Mild (≤5)	14 (43.8%)	13 (39.4%)	NS^a^
Moderate (6–10)	13 (40.6%)	19 (57.6%)	NS^a^
Severe (>10)	5 (15.6%)	1 (3%)	NS^a^
RQLQ, mean (Min - Max)	3.5 (1.83–5.77)	3.4 (1.52–5.1)	0.727
Total IgE, geometric mean kU/L (Mín - Máx)	698.9 (19.3–5.000)	502 (18.6–5.000)	0.466
Specific IgE, mean, kU_A_/L (Min - Max)			
Der p 1	29.5 (1.61–100)	25.9 (0.39–100)	0.682
Blo t	27.1 (1.43–100)	21.1 (2.01–100)	0.515
Specific IgG4, mean, kU_A_/L (Min - Max)			
Der p 1	0.13	0.08	0.09
Blo t	0.14	0.09	0.95
Comorbitidies			
Conjunctivitis (%)	26 (81.2%)	22 (66.7%)	NS
Asthma (%)	14 (43.7%)	17 (51.5%)	NS
Atopic dermatitis (%)	10 (31.2%)	14 (42.4%)	NS
Urticaria (%)	3.1%	0%	NS
Food allergy (%)	3.1%	0%	NS
Drug Hypersensitivity (%)	3.1%	3%	NS

N: absolute number; *p*: value-*p*; HDM SLIT: house dust mite sublingual immunotherapy; TNSS: total nasal symptoms score; RQLQ: quality of life questionnaire; Der p 1: *D. pteronyssinus* mite group 1 specific antigen; Blo t: *B. tropicalis* mite specific antigen; Min: minimum value; Max: maximum value; NS^a^ - Chi-square test

Other allergic conditions were observed among participants, and the more prevalent were allergic conjunctivitis (73.85%), asthma (47.69%), and atopic dermatitis (36.92%) (see Table 1). All patients diagnosed with asthma included in the study had been asymptomatic for 1 year or more, with FEV1 values greater than 70%, according to the inclusion criteria indicating that they were well-controlled.

In our study, 16.9% of participants were sensitized to aeroallergens other than *D. pteronyssinus* and *B. tropicalis*, but they did not experience direct exposure or clinical manifestations when exposed to those allergens. The clinical relevance of these sensitizations was determined based on patient history and exposure profiles. For instance, patients sensitized to cat dander who had a cat at home were excluded from this study.

### Efficacy of sublingual immunotherapy with *D. pteronyssinus* and *B. tropicalis* extracts in patients with allergic rhinitis

#### Use of medications to control symptoms

**Fig. 2:**
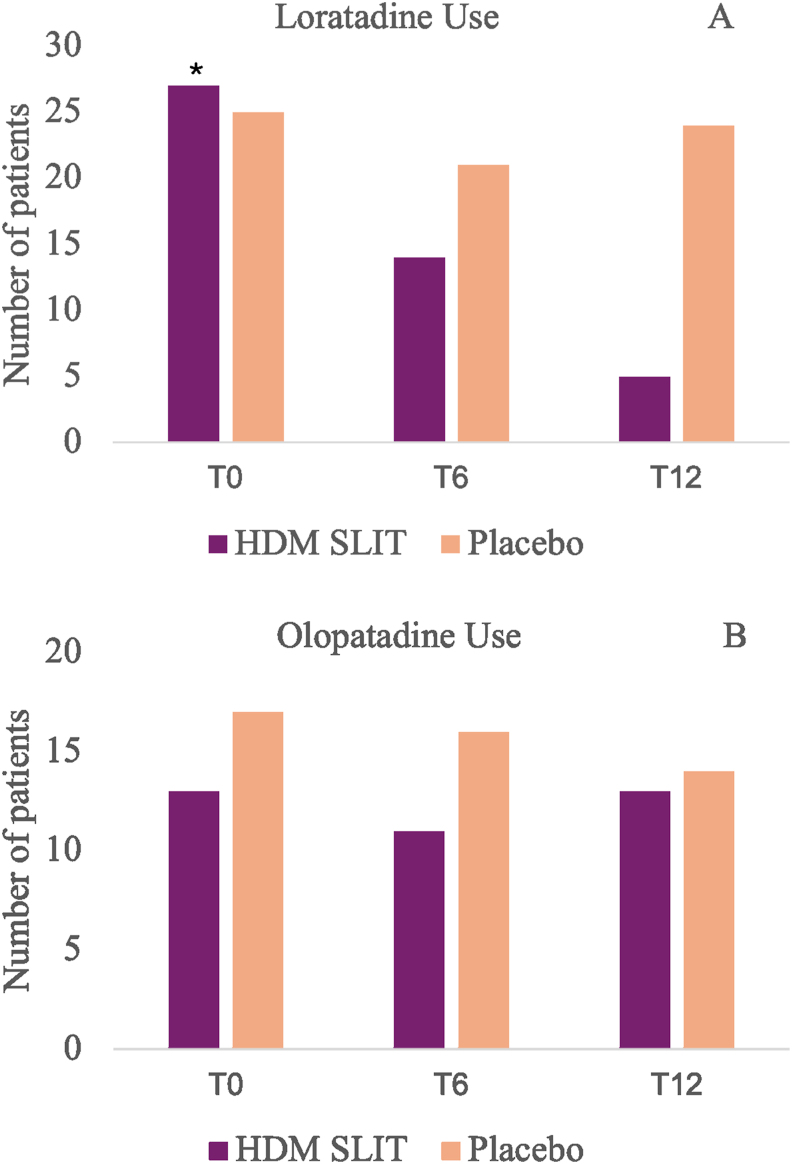
Comparison of the absolute number of patients between the HDM SLIT (house dust mite sublingual immunotherapy) and placebo groups at the baseline (T0) and at the end of the study (T12); 2A: comparison regarding the use of antihistamines (loratadine); 2B: comparison regarding the use of olopatadine; ∗p < 0.0001, Chi-square Test

At the beginning of treatment, 27 (81.8%) patients in the placebo group and 25 (78.1%) patients in the HDM SLIT group were using loratadine ≥ 3x/week. After 12 months, 24 patients (72.7%) were still using loratadine ≥3 times/week in the placebo group, but only 5 (15.6%) in the SLIT group. There was a statistically significant difference between the HDM SLIT and the placebo group for antihistamine consumption (p < 0.0001, Chi-square test) (Fig. 2A).

Among patients with AR-associated conjunctivitis, 13 (40.6%) patients in the HDM SLIT group and 17 (51.5%) patients in the placebo group were using olopatadine eye drops. After 12 months, this number decreased to 11 (34.4%) and 16 (48.5%), respectively. There was no statistical significance within either group (p: 0.206 and p = 0.285, McNemar test) or between groups (p = 0.248, chi-square test) (Fig. 2B).

**Fig. 3:**
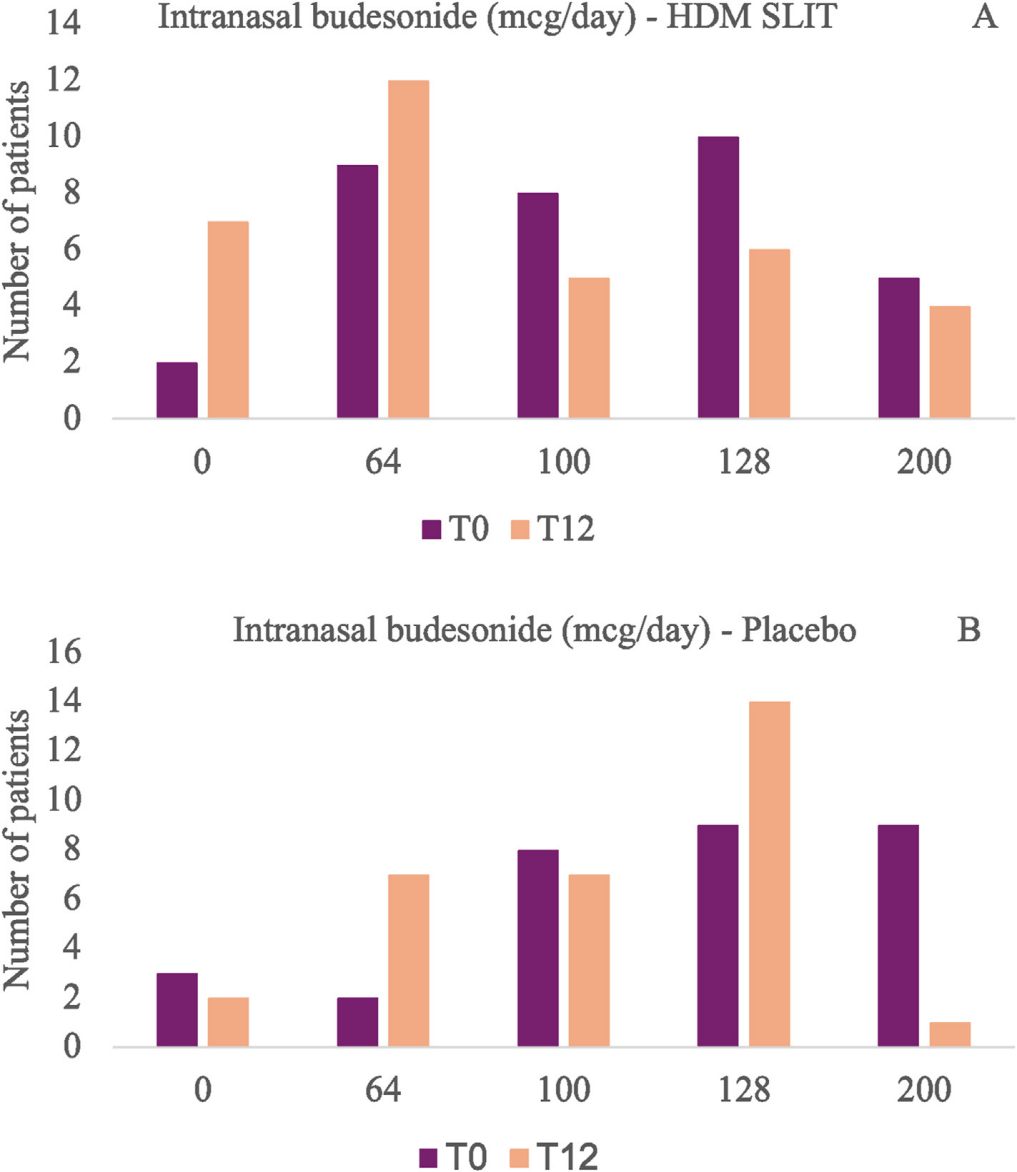
Comparison of budesonide dosage (mcg/day) between the HDM SLIT (house dust mite sublingual immunotherapy) and placebo groups at the baseline (T0) and at the end of the study (T12); 3A: HDM SLIT group; 3B: placebo group

After the twelve-month follow-up, there was a reduction in the average consumption of budesonide, both in the placebo group (10% reduction) and in the HDM SLIT group (19.7% reduction), with no statistical difference between groups (p = 0.26, Chi-square test) (Fig. 3A and B).

#### Symptom and quality of life scores

For RQLQ, the main quality of life activities most impacted by rhinitis symptoms were housework, playing with animals, using a vacuum cleaner, doing repairs at home, and undertaking physical activity (Supplement table I).

Table 2 summarizes the TNSS and RQLQ data. Both in the HDM SLIT group and in the placebo group, there was a reduction in the RQLQ value greater than 0.5, but without a statistically significant difference between the groups (p = 0.962, Mann-Whitney test). Regarding the nasal symptoms score, no statistically significant reduction in symptoms when comparing T0 and T12 between groups (p = 0.38, Mann-Whitney test) was observed.

**Table 2:** TNSS and RQLQ questionnaire values in SLIT and placebo groups over 12 months.

	Time	HDM SLIT (median)	Placebo (median)	*p*-value
RQLQ	T0	3.19 ± 1.05	3.27 ± 0.93	0.727
	T6	1.28 ± 1.14	1.71 ± 1.05	0.609
	T12	1.73 ± 1.14	1.67 ± 0.9	0.962
TNSS	T0	6.00 ± 3.04	6.00 ± 2.6	0.874
	T6	4.50 ± 3.07	5.50 ± 2.91	0.429
	T12	5.00 ± 3.08	6.50 ± 3.26	0.380

HDM SLIT: house dust mite sublingual immunotherapy; RQLQ: quality of life questionnaire; TNSS: total nasal symptoms score

#### Laboratory assessment

There was no significant difference in serum immunoglobulin levels between the groups at baseline (p > 0.05, Chi-square test) (see Table 1). In the analysis of the dynamics of total IgE, specific IgE, and IgG4 levels for both allergens, no significant differences were observed between the HDM SLIT and placebo groups (p > 0.05, Mann-Whitney test) – Supplement figure I.

#### Adverse events

Of the 65 patients who completed the study, 91 adverse events (AE) were reported in the first 6 months (T0-T6) and 54 adverse events in the last 6 months of the study (T6-T12).

The main adverse events were worsening of ocular symptoms, followed by dyspnea. Table 3 summarizes the adverse events and compares the data between the 2 evaluated periods (T0-T6 and T6-T12). Overall, adverse events were reported at similar frequencies between the HDM SLIT and placebo groups during both periods. There were no serious adverse events, such as anaphylaxis.

**Table 3:** Incidence of adverse events in SLIT and placebo groups in at the 6 months and 12 months.

Adverse events	T0 – T6			T6 – T12		
	HDM SLIT	Placebo	*p*-value	HDM SLIT	Placebo	*p*-value
Mouth ulcers	4	2	0.37	1	4	0.41
Itching in oropharynx	5	4	0.24	2	2	0.67
Dyspnea	4	5	0.11	4	4	0.52
Cough	3	4	0.72	3	1	0.29
Urticaria	3	2	0.62	3	0	0.07
AD worsening	5	8	0.78	2	5	0.25
Itching ear	2	2	0.97	1	2	0.57
Abdominal pain	3	3	0.62	0	1	0.32
Dysphagia	0	1	0.32	1	1	0.98
Nausea	0	1	0.32	0	0	0.90
Eyes symptoms	15	11	0.88	9	7	0.52
Total	44	47		27	27	

HDM SLIT: house dust mite sublingual immunotherapy; AD: atopic dermatitis; T0 – T6 baseline and at the 6 months; T6 – T12: 6 months and at the end of the study (12 months)

In the first month of the study, 9 patients complained of dyspnea between the fifth and tenth day of treatment. No patient needed to go to the emergency room. All were instructed to discontinue HDM-SLIT as soon as they reported symptoms and were called for evaluation. All were medicated with SABA for symptom relief, and only 1 patient needed to start using inhaled corticosteroids and was excluded from the study. After the study was unmasked, it was identified that this patient belonged to the placebo group. None of the patients needed to use oral corticosteroids.

Two patients complained of dysphagia (1 HDM SLIT and 1 placebo). Both underwent upper digestive endoscopy, and Eosinophilic Esophagitis was ruled out, evolving with clinical improvement and follow-up in the study.

## Discussion

In this study, we present the results of a randomized, double-blinded, placebo-controlled clinical trial investigating the safety and efficacy of HDM SLIT treatment in patients with moderate to severe allergic rhinitis (AR). For safety reasons, only patients with intermittent asthma were included in this trial. This is the first randomized double-blind placebo-controlled trial involving SLIT with *B. tropicalis*.^19^, ^20^, ^21^, ^22^

After 12 months of treatment, using a 1 mcg dose of Der p 1 associated with 753 UBE of Blo t/day, we identified a significant reduction in antihistamine consumption in the SLIT group and no significant difference in the placebo group. Although there was no significant reduction in budesonide consumption between the groups, like in other HDM SLIT trials,^22^^,^^29^ the reduction in antihistamine intake allows us to infer that after 1 year of treatment with HDM SLIT, the rhinitis outbreaks were better controlled than in the placebo group.

Regarding the TNSS and RQLQ, both groups exhibited a reduction in scores, but there was no minimal clinically significant difference between them. All patients were oriented to not change their medications without medical prescription. Regular visits and the use of pharmacotherapy may have influenced treatment adherence and the improvement scores in both assessments. Additionally, the involvement of only 2 researchers throughout the study likely affected the doctor-patient relationship, leading to a potential Hawthorne effect. This effect can alter perception of symptoms, with patients tending to minimize or deny their severity, resulting in lower TNSS and RQLQ scores.

Since the study was double-blinded, the researchers' assessments could be compared with patient-reported data, helping to mitigate the influence of this behavior. However, some patients had already been participating for a few months, making it impossible to include the researchers’ TNSS and RQLQ assessments. The placebo effect, inherent to the study design,^30^ may also contribute to the clinical benefits observed in the placebo group.

Although the European Academy of Allergy and Clinical Immunology (EAACI) has defined the Combined Symptom and Medication Score (CSMS) as the standard primary endpoint for AIT trials,^29^ this endpoint had not been validated at the start of our study.^31^^,^^32^ While we do not have CSMS data and observed no differences in the consumption of intranasal corticosteroids or symptom scores, the 50% reduction in oral antihistamine intake within the SLIT group suggests that the proportion of patients experiencing rhinitis exacerbations was significantly lower with HDM SLIT drops. Additionally, we selected the TNSS to assess the severity of rhinitis symptoms. However, since the TNSS focuses solely on nasal symptoms and does not include ocular symptom assessment, we were unable to evaluate the CSMS.

To evaluate the immunological response, we measured serum levels of total IgE, specific IgE, and specific IgG4 for Der p 1 and Blo t. In the first 6 months, we observed a decrease in both specific IgE and IgG4 levels for both allergens in the SLIT group, followed by an increase between 6 and 12 months. By contrast, no such changes occurred in the placebo group. Although the serum levels of IgE for Der p 1 showed an initial reduction followed by an increase, this latter increase was not statistically significant. However, reduced loratadine consumption in the HDM SLIT group after 6 months, coupled with the decrease in specific IgE and IgG4 levels for Der p 1 and Blo t*,* suggests that IgG4 may play a role in blocking IgE and thus suppressing histamine release from basophils.

The literature presents varied results regarding the dynamics of specific IgE and IgG4 levels. Some studies report decreases, increases, or unchanged levels of specific IgE,^32^, ^33^, ^34^, ^35^, ^36^ while findings for specific IgG4 levels vary as well, with some studies indicating increases and others showing no variation.^34^^,^^35^ Notably, other research has found elevated specific IgG4 levels only after 18 months of treatment.^37^ The isolated levels of specific IgG4 have been debated as a reliable biomarker for AIT efficacy at the individual level. In contrast, evaluating functional IgG4 measurements appears to be a more promising approach.^36^, ^37^, ^38^ Our group has conducted studies analyzing functional measures of specific IgG4 in some of these subjects, revealing an increase in the diversity of IgG4 epitopes, which indicates greater binding capacity of the IgG4 antibody to allergenic peptides.^39^

The safety of the HDM SLIT-D mix was evaluated based on the number and severity of the adverse events (AEs) observed. Like other studies, the frequency of adverse events was highest at the study's outset.^40^ There was no significant difference between the groups in the number of adverse events or severe adverse events/anaphylaxis. At the beginning of this trial, 9 patients reported dyspnea and dry cough (5 from the SLIT group and 4 from the placebo group). None of the patients required oral corticosteroids; they only used short-acting beta-agonists (SABAs) to relieve their symptoms, and none needed to visit the emergency room. When they reported these symptoms, we advised them to suspend sublingual treatment and scheduled evaluations for further assessment and care. Even after reducing the administered dose, we maintained a dose higher than the typical SLIT dosage used routinely in our country.

Therefore, we can infer that the dose used, particularly 753 UBE of Blomia, was safe. However, more studies with scaling doses for *D. pteronyssinus* and *B. tropicalis* sublingual immunotherapy are necessary to determine the ideal dose for our population, considering the particularities of tropical and subtropical countries.

There were few studies for *B. tropicalis* involving either SLIT or SCIT.^19^, ^20^, ^21^, ^22^, ^23^ Most clinical trials with SLIT and HDM only evaluate *D. pteronyssinus* and *D. farinae*. This occurs mainly because most studies were conducted in temperate climate countries, presenting low *B. tropicalis* prevalence. In tropical regions like Brazil, the prevalence of sensitization to *B. tropicalis* is high,^17^ and unlike *D. farinae* allergens, *B. tropicalis* allergens show low homology with *D. pteronyssinus*.^19^ Because of this, some researchers suggest the inclusion of *B. tropicalis* in the preparation of AIT.^18^^,^^41^

In Brazil, for example, the prevalence of sensitization in patients with allergic rhinitis is around 83.2% for *D. pteronyssinus* and 70.3% for *B. tropicalis*.^16^

As the prevalence of sensitivity to *B. tropicalis* is high, combining *B. tropicalis* extract with *D. pteronyssinus* in preparing AIT is a common practice between us, as SCIT as SLIT. Regarding *D. pteronyssinus*, in SCIT, based on European studies, a dose of 5–20 mcg/month of major *D. pteronyssinus* allergens is used;^42^ and, in some patients, it is necessary to reduce the dose to continue the treatment. Optimal SLIT doses have been determined only for SLIT tablets from randomized, double-blind, placebo-controlled dose-finding clinical trials, with a dose of 300IR considered safe and effective.^15^ To date, the dose of SLIT-D needs to be better defined. Despite the similarity in administration route, that is, sublingual, there are differences in physicochemical characteristics between the tablet formulation and drops.^14^ According to AAAAI/ACAAI 2017, it is not possible to affirm that effective SLIT-D is equivalent to SLIT-T dosing.^43^ Since there is no consensus regarding the dose for SLIT-D, in practice, North American allergists have used the maintenance dose for SCIT based on major allergen content, suggesting that the daily dose of SLIT-D should be equivalent to the monthly dose of SCIT.^14^ In Brazil, unlike North American practice, until the beginning of this study, the dose used was lower than the dose considered effective.^44^ The dose used by Brazilian allergists was 0.15 mcg of significant allergens of Der p/day (being 0.06 mcg of Der p 1).

Nevertheless, although considered a low dose, a local study not controlled by placebo showed an excellent clinical response.^45^ In another study, the authors draw attention to the particularities of allergies in tropical regions and the need for further research given the climatic, dietary and demographic diversity.^46^ Given the results of this local study and the climatic differences in the tropics, with more humid, hotter weather (with higher dust mite load), we question whether the dose used in temperate countries would be safe for tropical countries. We used a Der p 1 dose 16 times smaller than the daily maintenance dose indicated as safe and effective from SLIT-T^15^^,^^47^ and 5 times lower than the monthly dose used in SCIT in the practice of Brazilian allergists.

## Summary

Although there is a dose-dependent effect in the immune and clinical response and a low dose of Der p 1 was used, when compared to the literature, the results of this study demonstrated that, after 1 year, SLIT drops containing 1 mcg of Der p 1/day and 753 UBE of Blo t/day promoted a decrease in oral antihistamine intake, indicating a better control of rhinitis exacerbations for the placebo group. Given the environmental differences and genetic characteristics between tropical and temperate countries, further research is needed to evaluate the ideal SLIT dose, as well as the specifications for the formulation adopted (drops or tablets).

Regarding the *B. tropicalis* dose, unfortunately, in the literature, the only 2 articles using *B. tropicalis* extract in SLIT employed different measurements to quantify the dose. We used the dose of 753 UBE/day already adopted in the practice of Brazilian allergists, but instead of 3 times a week (9036 UBE Blo t/month), daily use was employed (22,590 UBE/month), resulting in a cumulative monthly dose 2.5 times higher. Although the sample size in this study was small, there were no serious adverse events seen in patients receiving 753 UBE of Blo t/day. More studies are needed to define the ideal dose in SLIT drops.

## Abbreviations

**AAAAI**, American Academy of Allergy, Asthma and Immunology; **ACAAI**, American College of Allergy, Asthma and Immunology; **AE**, Adverse Event; **AIT**, Allergen immunotherapy; **AR**, allergic rhinitis; **ARIA**, Allergic Rhinitis and its Impact on Asthma; **ASBAI**, Associação Brasileira de Alergia e Imunologia; ***B. tropicalis***, *Blomia tropicalis* mite; **Blo t**, *Blomia tropicalis* antigen; **CSMS**, Combined Symptom and Medication Score; **D. farinae**, *Dermatophagoides farinae* mite; **D. pteronyssinus**, *Dermatophagoides pteronyssinus* mite; **Der p 1**, *Dermatophagoides pteronyssinus* antigen 1 (major HDM allergen); **Der p 2**, *Dermatophagoides pteronyssinus* antigen 2; **Der f**, *Dermatophagoides farina* antigen; **EMA**, European Medicine Association; **EAACI**, European Academy of Allergology and Clinical Immunology; **FDA**, Food and Drug Administration; **FEV**_**1**_, forced expiratory volume in first second; **HDM**, house dust mite; **HDM SLIT**, house dust mite sublingual immunotherapy; **IgA**, Immunoglobulin A; **IgA1**, Immunoglobulin A subtype 1; **IgA2**, Immunoglobulin A subtype 2; **IgE**, Immunoglobulin E; **IgG4**, Immunoglobulin G subtype 4; **ISAAC**, International Study on Asthma and Allergy in Childhood; **RDBPC**, andomized, double-blind, placebo-controlled; **RDBPCT**, Randomized, double-blind, placebo-controlled trial; **RQLQ**, rhinoconjunctivitis quality of life questionnaire; **SCIT**, subcutaneous immunotherapy; **SLIT**, sublingual immunotherapy; **SLIT-D**, SLIT administered by drops; **SLIT-T**, SLIT administered by tablets; **TNSS**, Total Nasal Symptom Score.

## Funding sources

The laboratory IPI-ASAC Brasil/ASAC Pharma Brazil provided the mite extracts for immunotherapy. Thermo Fisher Scientific, Sweden, provided the laboratory reagents for IgE and IgG4 determination. The funding sources had no role in the study design, collection, analysis, data interpretation, report writing, and the decision to submit the article for publication.

## Data availability statement

All data generated or analyzed during this study are included in this article. Further enquiries can be directed to the corresponding author.

## Authors’ consent for publication and author contributions

All the above authors have contributed substantially to the conception and design of the study, the acquisition, analysis, and interpretation of data. They all have contributed with the article drafting and have revised it critically for important intellectual content. All the authors have gave their final approval of the submitted version and have gave their consent for publication in case it is accepted.

## Statement of ethics

The trial was conducted in accordance with the Declaration of Helsinki and the International Conference on Harmonization Good Clinical Practice guidelines. The Ethics Committee of the University of Sao Paulo Medical School approved this study (protocol no. 1.978.680). Each subject signed assent and written informed consent and, when appropriate (subjects under 18 years old), by parents or legal guardians.

## Declaration of competing interest

The authors have no conflicts of interest to declare.

## Figures and Tables

**Figure:**
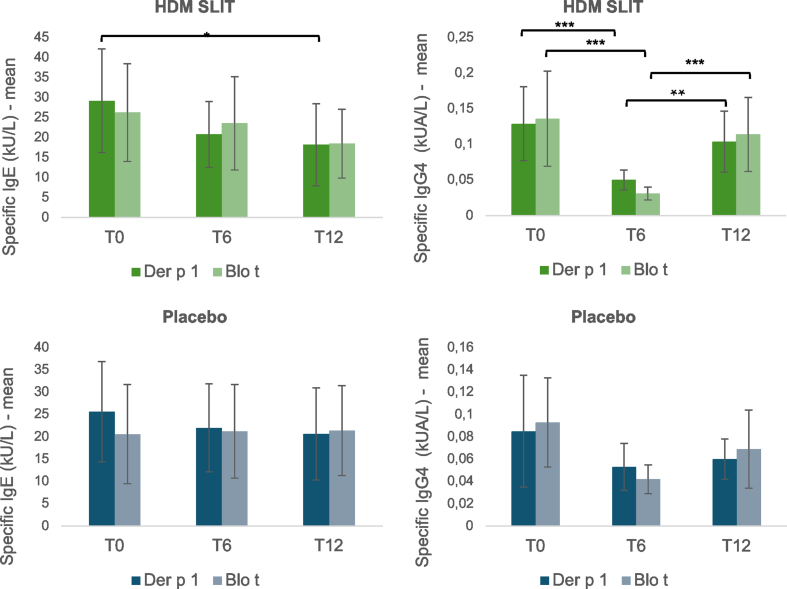
FigS1Evolution of serum levels of specific IgE and IgG4 in the HDM SLIT (house dust mite sublingual immunotherapy) and placebo groups, at times T0, T6 and T12.4 A: serum IgE levels for Der p 1 and Blo t, in the HDM SLIT group; 4B: serum IgG4 levels for Der p 1 and Blo t, in the HDM SLIT group; 4C: serum IgE levels for Der p 1 and Blo t, in the placebo group; 4D: serum levels of IgG4 for Der p 1 and Blo t, in the placebo group; ∗p < 0.05; ∗∗p < 0.01; ∗∗∗p < 0.001, ANOVA test.
