# Supplementary material for: Efficacy and safety of sublingual immunotherapy using a combination of Dermatophagoides pteronyssinus and Blomia tropicalis extracts in patients with allergic rhinitis: A randomized, double-blind, placebo-controlled trial
Source: World Allergy Organ J. 2025 Jan 28;18(2):101020. doi: 10.1016/j.waojou.2024.101020 (PMC11815642; doi:10.1016/j.waojou.2024.101020)
Supplement: Supplementary file 1 — Multimedia component 1 [file mmc1.doc]

Supplemental table 1: RQLQ activities most selected by the 65 patients who completed 1 year of treatment.

| Activities | HDM SLIT | Placebo | TOTAL |
| --- | --- | --- | --- |
| Housework | 19 | 21 | 40 |
| Playing with pets | 8 | 9 | 17 |
| Vacuuming | 5 | 11 | 16 |
| Home maintenance | 7 | 8 | 15 |
| Exercising or working out | 5 | 6 | 11 |
| Reading | 6 | 3 | 9 |
| Using computer | 5 | 2 | 7 |
| Going for walking | 3 | 4 | 7 |
| Sports | 3 | 3 | 6 |
| Watching TV | 3 | 2 | 5 |
| Visiting friends and relatives | 1 | 4 | 5 |
| Outdoor activities | 2 | 3 | 5 |
| Social activities | 2 | 2 | 4 |
| Eating | 3 | 1 | 4 |
| Gardening | 3 | 0 | 3 |
| Iron | 3 | 0 | 3 |
| Taking care of the dog | 3 | 0 | 3 |
| Work-related activities | 1 | 2 | 3 |
| Swimming | 1 | 1 | 2 |
| Mowing the lawn | 0 | 2 | 2 |
| Talking | 1 | 1 | 2 |
| Bicycling | 0 | 1 | 1 |
| Playing with children | 1 | 0 | 1 |
| Driving | 1 | 0 | 1 |
| Singing | 1 | 0 | 1 |
| Going to the park | 0 | 1 | 1 |
| Shopping | 0 | 0 | 0 |
| Play tennis | 0 | 0 | 0 |
| Sitting outdors | 0 | 0 | 0 |

HDM SLIT: house dust mite sublingual immunotherapy
