# Supplementary material for: Efficacy and safety of sublingual immunotherapy using a combination of Dermatophagoides pteronyssinus and Blomia tropicalis extracts in patients with allergic rhinitis: A randomized, double-blind, placebo-controlled trial
Source: World Allergy Organ J. 2025 Jan 28;18(2):101020. doi: 10.1016/j.waojou.2024.101020 (PMC11815642; doi:10.1016/j.waojou.2024.101020)
Supplement: Multimedia component 2 [file mmc2.docx]

Anexo 1: Sublingual immunotherapy schedule

- 1th day: 1 drop
- 2th day: 2 drops
- 3th day: 3 drops
- 4th day: 4 drops
- From the 4th day: 4 drops every day

HOW TO USE:

- - Do not administer the medication on an empty stomach.
  - Place the drop(s) under the tongue and only swallow after 1 minute of swallowing.
  - Do not eat or drink 5 minutes after administration.
  - If adverse reactions (listed in the action plan) 🡪 follow ACTION PLAN guidelines
  - If you forget 1 dose DO NOT DO a double dose the next day  write down forgotten dose
  - Keeping the vaccine in the refrigerator - not keeping the vaccine in the refrigerator means reducing the potency of the extract, impairing the treatment.

Anexo 2: Action Plan

**ACTION PLAN FOR LOCAL AND SYSTEMIC REACTIONS WITH SUBLINGUAL IMMUNOTHERAPY**

Patient: ____________________________________________ Age: _______

Allergies: _______________________________________________________

Additional health problems: _________________________________________

Concurrent medications:____________________________________________

**FOR SEVERE LOCAL REACTION***

• Mouth/Throat: swelling that causes hoarseness and/or throat closing

• OR FOR SYSTEMIC REACTION*

• Skin: hives all over body and/or redness all over body

• Lung: shortness of breath, cough, and/or wheezing

• Heart: weak pulse, dizziness, and/or passing out

• Gastrointestinal: severe abdominal pain. Vomiting, diarrhea, and/or cramping

*** You may only have a few symptoms. Symptoms can be life-threatening.**

**ACTION 🡪 Inject epinephrine in thigh using (circle one):**

• Epinefrin 0.3 mg

Contact of emergency: Phone of the researcher

**FOR MILD TO MODERATE LOCAL REACTION**

•Mouth: bothersome itching, and/or mild swelling of lips and/or tongue

• Throat: bothersome itching, irritation, and/or mild tightness

• Ear: bothersome itching

• Gastrointestinal: mild abdominal pain, nausea, and/or cramps

**ACTION 🡪** Use Antihistamine: loratadine 10 mg

If wheezing, dyspnea, shortness of breath: ___ puffs de salbutamol (100 mcg/puff).

Adapted of Epstein et al, 2016
